# Development of a mobile application to represent food intake in inpatients: dietary data systematization

**DOI:** 10.1186/s12911-023-02406-x

**Published:** 2024-01-30

**Authors:** Alan Renier Jamal Occhioni Molter, Naise Oliveira da Rocha Carvalho, Paloma Ribeiro Torres, Marlete Pereira da Silva, Patrícia Dias de Brito, Pedro Emmanuel Alvarenga Americano do Brasil, Claudio Fico Fonseca, Adriana Costa Bacelo

**Affiliations:** 1 Evandro Chagas National Institute of Infectious Diseases, the Oswaldo Cruz Foundation, Rio de Janeiro, RJ Brazil; 2 Veiga de Almeida College, Rio de Janeiro, RJ Brazil; 3 Grupo de Pesquisa Clínica Em Nutrição E Doenças Infecciosas, Instituto Nacional de Infectologia, Fundação Oswaldo Cruz, Rio de Janeiro, CEP 21040-360 Brazil

**Keywords:** Diagnosis, Malnutrition, Dietetics, Monitoring, mHealth, Health app

## Abstract

**Background:**

Nutritional risk situations related to decreased food intake in the hospital environment hinder nutritional care and increase malnutrition in hospitalized patients and are often associated with increased morbidity and mortality. The objective of this study is to develop and test the reliability and data similarity of a mobile application as a virtual instrument to assess the acceptability and quality of hospital diets for inpatients.

**Methods:**

This intra- and interobserver development and reliability study investigated an in-hospital food intake monitoring application based on a validated instrument for patients with infectious diseases who were treated at the Evandro Chagas National Institute of Infectious Diseases (INI/FIOCRUZ). The instrument was sequentially administered to patients 48 h after admission to INI hospital units using the printed instrument (paper) and the digital application (ARIETI) simultaneously. The tested reliability factor was the consistency of the method in the digital platform, checking whether the application provided equivalent data to the paper instrument, and finally, a statistical analysis plan was performed in the R platform version 4.2.0. This project was authorized by the FIOCRUZ/INI Research Ethics Committee.

**Results:**

The ARIETI was developed and tested for reliability in 70 participants, showing a similar ability to calculate caloric intake in Kcal, protein intake (g), the proportion of caloric intake and protein intake relative to the prescribed goal. These instrument comparison analyses showed statistical significance (*p* < 0.001). The application was superior to the paper-based instrument, accelerating the time to perform the nutritional risk diagnosis based on food intake by approximately 250 s (average time).

**Conclusions:**

The ARIETI application has demonstrated equivalent reliability compared to the original instrument. Moreover, it optimized the time between the diagnosis of nutritional risk related to dietary intake and the nutritionist’s decision making, showing an improved ability to maintain information quality compared to the paper-based instrument.

## Background

Malnutrition is an important public health problem worldwide. The risk of decreased food intake in the hospital environment hinders nutritional care, representing an important malnutrition factor [1], increasing the morbidity rate and extending the hospital stay [2]; thus, food intake is crucial for analyzing the patient’s nutritional, clinical, and hospital conditions.

Factors such as the underlying disease, coexisting acute or chronic comorbidities, medication side effects, physical inactivity, food supply deficiency and/or intake, psychological factors, inappetence or loss of the sense of taste, inability to ingest food or malabsorption, and the hospital environment itself, as well as frequent negligence of health teams regarding nutritional aspects to the detriment of other cars, can influence the nutritional status before, during, and even after hospitalization [3, 4].

Proper monitoring of dietary acceptance ensures correct nutrient intake to hospitalized patients and can preserve or recover their nutritional status [5]. Patients with infectious diseases such as AIDS, tuberculosis, Chagas and paracoccidioidomycosis, and coronavirus disease (COVID), among others, have increased nutritional demand and impaired food intake capacity as a result of inflammatory cascades [6, 7].

Few validated instruments for monitoring in-hospital food intake can be quickly administered. However, Silva [8] developed a consistent instrument with a good correlation with the weighing method, the gold standard for dietary anamnesis, which requires no training in nutrition, only being literate [8]. Despite these advantages, as this instrument is structured in paper, it carries the risk of document loss and transcription and/or analysis error regarding prescription [9]. Moreover, using this instrument, the nutritionist has to manually calculate both the caloric and protein intake (PTN) and adapt it to the prescription and patients’ nutritional needs to enable technical interpretation, risk diagnosis, and prescribed conduct adjustments.

To date, we have not identified an application or study that presents a validated digital instrument for the analysis of nutritional risk in a hospital setting. The applications identified in the literature and the market focus solely on assessing quality of life, such as weight reduction and diabetes control. Thus, the development of a digital solution based on Silva’s tool [8] simplifies and accelerates the diagnosis of in-hospital dietary intake by automatically calculating the food intake and the degree of adequacy or inadequacy according to the patient’s nutritional needs without the need for additional calculations and with greater information security.

The objective of this study is to develop and test the reliability and data similarity of a mobile application as a virtual instrument to assess the acceptability and quality of hospital diets for inpatients.

## Methods

### Development

A digital solution for the diagnosis of nutritional risk based on in-hospital dietary intake was developed using multiplatform programming software, including Android and later IOS, in combination with C# and JSON technologies due to the high representativeness of the Android mobile platform in the Brazilian and global consumer market [10].

**Fig. 1:**
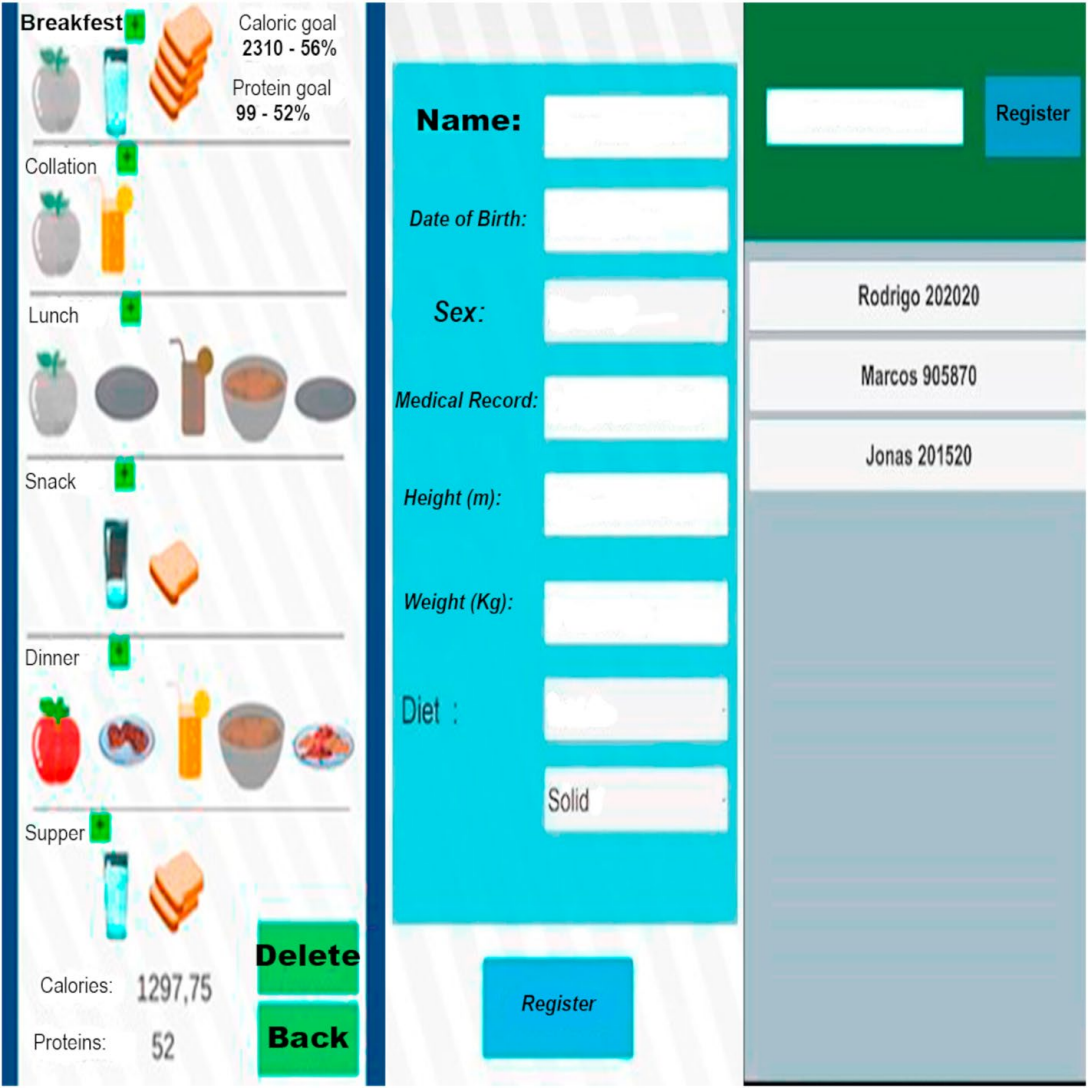
Application registration and data collection screens

The digital solution developed was termed “Mobile Application to Represent the Food Intake of Inpatients” (ARIETI; Aplicativo móvel de Representação da Ingestão alimEntar de pacienTes Internados). The ARIETI is an improvement of the analog instrument (on paper) proposed by Silva [8] while still maintaining the humanized design and practical usability of the original instrument (Fig. 1).

The ARIETI was developed to identify variations in food intake calories, proteins, carbohydrates, and lipids and to consider diet consistency variations, the dietary restrictions planned in the patient’s menu (sucrose, protein, or fats), and the partial or full acceptance of the different components of large and small meals (fruit, milk, bread, rice, beans, etc.), correlating them with the established therapeutic proposal (prescribed diet) and the patient’s nutritional goal (caloric and protein nutritional needs). The aim of the ARIETI was to help the nutritionist identify nutritional risks related to inadequate food intake and to highlight the percentage of calorie or protein inadequacy and the reasons reported for the inability of partial or global intake to identify the need for dietary prescription adjustments. The ARIETI also optimizes the physical space for information storage, provides data security, and streamlines communication to minimize the prevalence of hospital malnutrition.

The ARIETI is an application (health app) that was developed using Unity Software (Unity version 2019.1.1f1 (64-bit), 2019), which promotes more reliable data collection by making it simple and intuitive and can be easily used by any professional. ARIETI reduces the possibility of human calculation errors by aggregating all tools and algorithms, which optimizes the diagnosis of nutritional risk related to insufficient food intake, which is essential for more assertive decision making by the nutritionist.

### Study design and location

This observational, prospective study conducted a development and reliability assessment of a technological innovation tool for in-hospital dietary anamnesis and was approved by the FIOCRUZ/INI Research Ethics Committee (CAAE No. 35379820.4.0000.5262). The tool was tested in patients hospitalized at the FIOCRUZ/INI. The informed consent was obtained from all subjects and/or their legal guardian. The research was conducted considering Resolution No. 466, dated December 12, 2012, which approves the following guidelines and regulatory standards for research involving human subjects in Brazil.

#### Inclusion and exclusion criteria

The inclusion criteria were inpatients diagnosed with infectious disease who were awake, oriented and able to answer the instrument coherently, aged between 19 and 65 years, of both sexes, and who agreed to participate in the study.

The exclusion criteria were in patients who were comatose or disoriented, with any diagnosed psychiatric disorder that could affect the coherence of the responses to the instrument, or with confirmed infectious disease in the first 24 h of hospitalization.

### Data collection

The food intake data of the participants were simultaneously collected in the printed form (Silva’s analog instrument, 2017) and in the ARIETI by two research collaborators who had experience in dietary assessment.

The time of bedside dietary assessment and the time between the inclusion of the participants’ responses and the diagnosis of nutritional risk secondary to the dietary intake pattern in the previous 24 h, both reported on paper and in the ARIETI, were entered into the RedCap platform. This was implemented to reduce data quality issues, allow dynamic data management [11], help ensure control and confidentiality, and provide efficient data transfer into statistical analysis software such as R-Project®.

The data collected by the ARIETI were stored on a MySQL server with a Hyper Text Transfer Protocol Secure (HTTPS) data encryption protocol to ensure protection; this protocol was chosen to protect the integrity and confidentiality of the data stored between the user’s hardware and the online application. All stored data are protected by the Transport Layer Security (TLS) protocol, which adds three additional layers of protection, including encryption, impossibility of corrupting data during transfer, and authentication, which ensures direct communication with the correct online application [12].

Recording food intake in the ARIETI allows a backup using an internet connection through the MySQL Workbench or using a USB platform between a computer, notebook, or tablet, thus enabling external storage and the creation of a functional relational database.

**Fig. 2:**
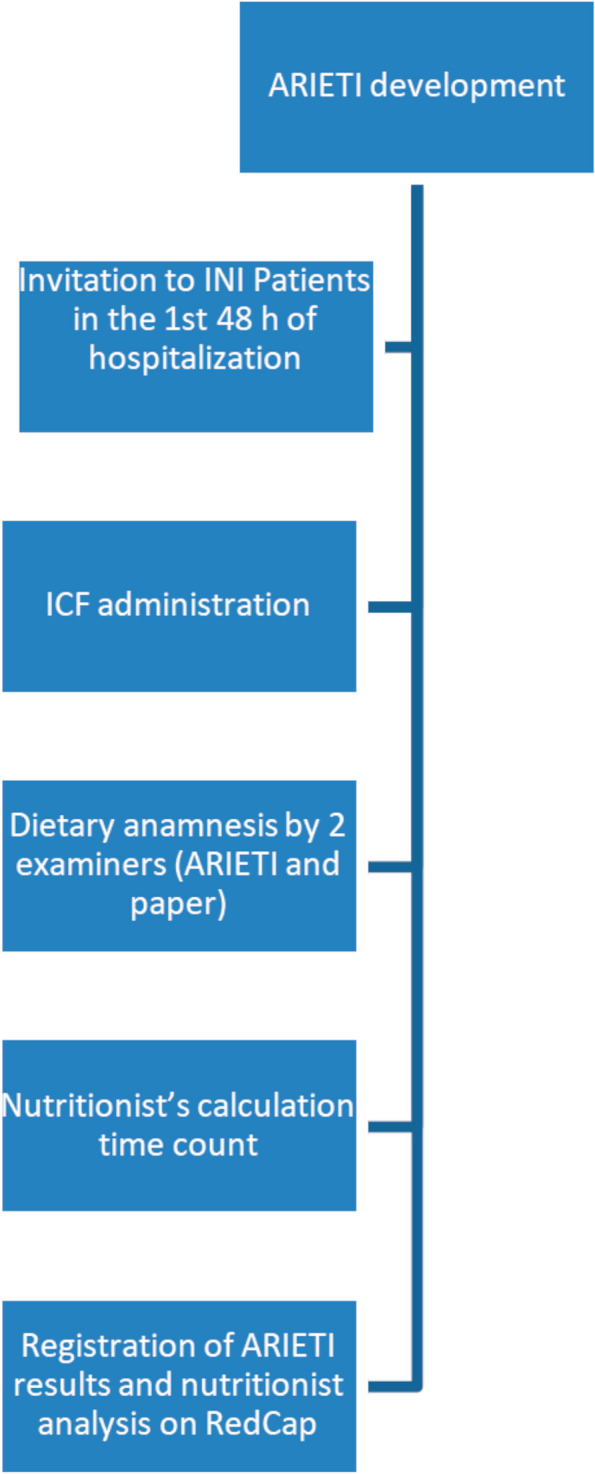
Flowchart of the study procedures

The data collection was carried out between March and October 2020. A sample size of 70 participants was used for the reliability test, considering a power of 0.8, and significance was determined if the *p* value was ≤ 0.001. The study procedures are illustrated in Fig. 2.

Embedded text: ARIETI development/Invitation to INI patients in the first 48 h of hospitalization/ICF administration/Dietary anamnesis by two examiners (ARIETI and paper)/Nutritionist’s calculation time count/Registration of ARIETI results and nutritionist analysis on RedCap. ARIETI: Aplicativo móvel de Representação da Ingestão alimEntar de pacienTes Internados; INI: Evandro Chagas National Institute of Infectious Diseases; ICF: informed consent form.

The reliability was verified by comparing the results of the dietary anamnesis between the paper and the ARIETI instruments. Brazilian hospitals use the Brazilian Table of Food Composition (TACO) as a reference for nutritional composition, with protein-to-calorie values being the main parameters. Protein intake is important due to its structural role in various tissues in the body. Measuring the amount of calories consumed is of utmost relevance given its involvement in various biochemical processes in the organism. Therefore, evaluating these variables is an important factor associated with food risk. Therefore, in addition to data on food intake and adequacy results calculated by the algorithm, the ARIETI organizes information including name, age, sex, weight, height, calorie requirements (Kcal/kg of weight), and protein (g/kg of weight), according to the clinical decision of the prescribing nutritionist.

**Fig. 3:**
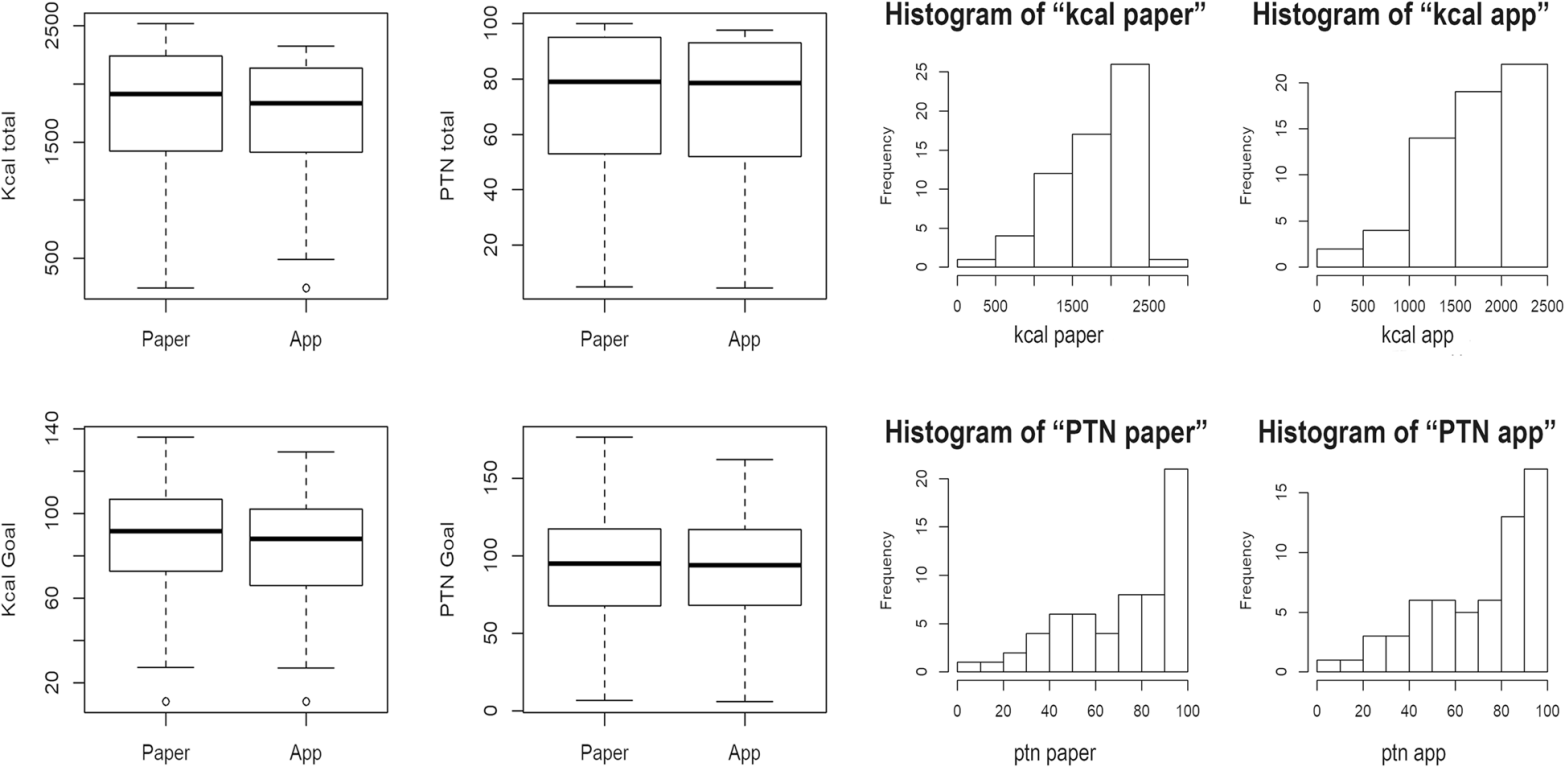
Histograms and boxplots showing the comparison of calorie and protein intake between the paper instrument and the ARIETI

The data compared between the instruments included dietary data, anamnesis time (filling out the paper instrument and the ARIETI), and the time between the start of the bedside assessment and the clinical decision making (Fig. 3).

The data were grouped by the following continuous quantitative variables, which were analyzed in both the paper and ARIETI:

- Total Kcal: Total number of kilocalories ingested by the patient in 24 h of hospitalization.
- Total PTN: Total amount of protein ingested by the patient over 24 h of hospitalization.
- Total Kcal goal: Percentage of the nutritional goal of the total number of kilocalories to be consumed by the patient, as stipulated by the nutritionist according to the patient’s nutritional status.
- Total PTN goal: Percentage of the nutritional goal of the total amount of protein to be consumed by the patient, as stipulated by the nutritionist according to the patient’s nutritional status.
- Time diff paper ARIETI: Difference between the administration time and decision making for the two instruments.

### Statistical analysis plan

The data distribution was assessed using histograms and the Shapiro–Wilk test. The analysis of normality and the statistical curve trend guided the selection of a hypothesis test (Pearson’s R and Wilcoxon’s test) to evaluate the agreement between the printed instrument and the ARIETI (health app). The Pearson coefficient only informs us about the existence of a linear relationship between the two variables and does not specify whether the relationship is causal. On the other hand, the Wilcoxon test does not require the original populations to be normally distributed or for their variances to be equal. However, it assumes that the distributions have a similar overall shape. This test assesses the null hypothesis that the medians of the values obtained from the two instruments are identical.

Data modeling was performed to compare the completion time and decision making. The Bland–Altman plot was used for visual comparison of the data.

All data will be analyzed using R-Project® software version 4.2.0, and differences was considered significant at a power of 0.8 if the *p* value is ≤ 0.001.

## Results

The calorie and protein intake recorded on paper and in the ARIETI were similar, as shown in the histogram with trend curves to the right in both instruments for both calorie and protein intake and in the boxplot, which shows a slightly smaller amplitude and median total calorie in the ARIETI than in the paper version (Fig. 3). The values of calories and Proteins obtained by the two instruments are shown in Table 1.

**Table 1:** Means and standard deviation of protein intake (g), number of calories, and percentage of nutritional goals achieved calculated by the two instruments

Variables	Paper mean	Paper SD	ARIETI mean	ARIETI SD
Kcal	1806.67	530.46	1717.87	517.64
PTN (grams)	71.86	24.94	70.55	24.47
Kcal goal	87.51	26.65	83.14	25.81
PTN goal (grams)	95.16	36.25	93.34	35.46

The outliers observed in the proportion of food intake capacity regarding calorie goals calculated by the ARIETI were the same as those calculated manually by the nutritionists.

No statistically significant difference was observed between the two instruments (paper-based and ARIETI) in relation to the dietary anamnesis variables examined for identifying nutritional risk associated with food intake. This demonstrates that the diagnostic outcomes generated by the paper instrument and the ARIETI are the same (Table 2).

**Table 2:** The results of the statistical tests confirm the similarity of the data obtained by both instruments (*p* < 0.001)

Variables	Statistics	*P* value	Hypothesis test
Kcal	1429.0	< 0.001	Wilcoxon test
PTN	1023.5	< 0.001	Wilcoxon test
Kcal goal	1492.0	< 0.001	Wilcoxon test
PTN goal	1119.0	< 0.001	Wilcoxon test
Kcal	1429.0	< 0.001	Pearson’s R
PTN	1023.5	< 0.001	Pearson’s R
Kcal goal	1492.0	< 0.001	Pearson’s R
PTN goal	1119.0	< 0.001	Pearson’s R

The *p* values were < 0.001 (Table 2) and relevant considering the values obtained from the analysis of pairs of the same variable between the two instruments regarding food intake of kilocalories, proteins, and their respective nutritional goals for Wilcoxon’s test and Pearson’s correlation. These results prove the statistically similar performance of the application compared to the paper instrument in all variables analyzed.

The Bland–Altman plot demonstrates the calculated mean of the two instruments and the measurement differences between them. The black line represents the mean nutritional goals and caloric and protein intake differences between the two instruments, while the two red dotted lines represent the 95% confidence interval limits for the mean difference. Figure 5 illustrates the linearity and equivalence of the data from both instruments, as they closely follow the trendline.

This graphic visualization method shows that data dispersion follows the mean differences between the variables studied with the two instruments used, following the expectancy for the trend line in the comparison between them. The dispersion showed a low number of outliers, with the data respecting their statistical equivalence, i.e., the instruments represent food intake with the same quality and capacity.

**Fig. 4:**
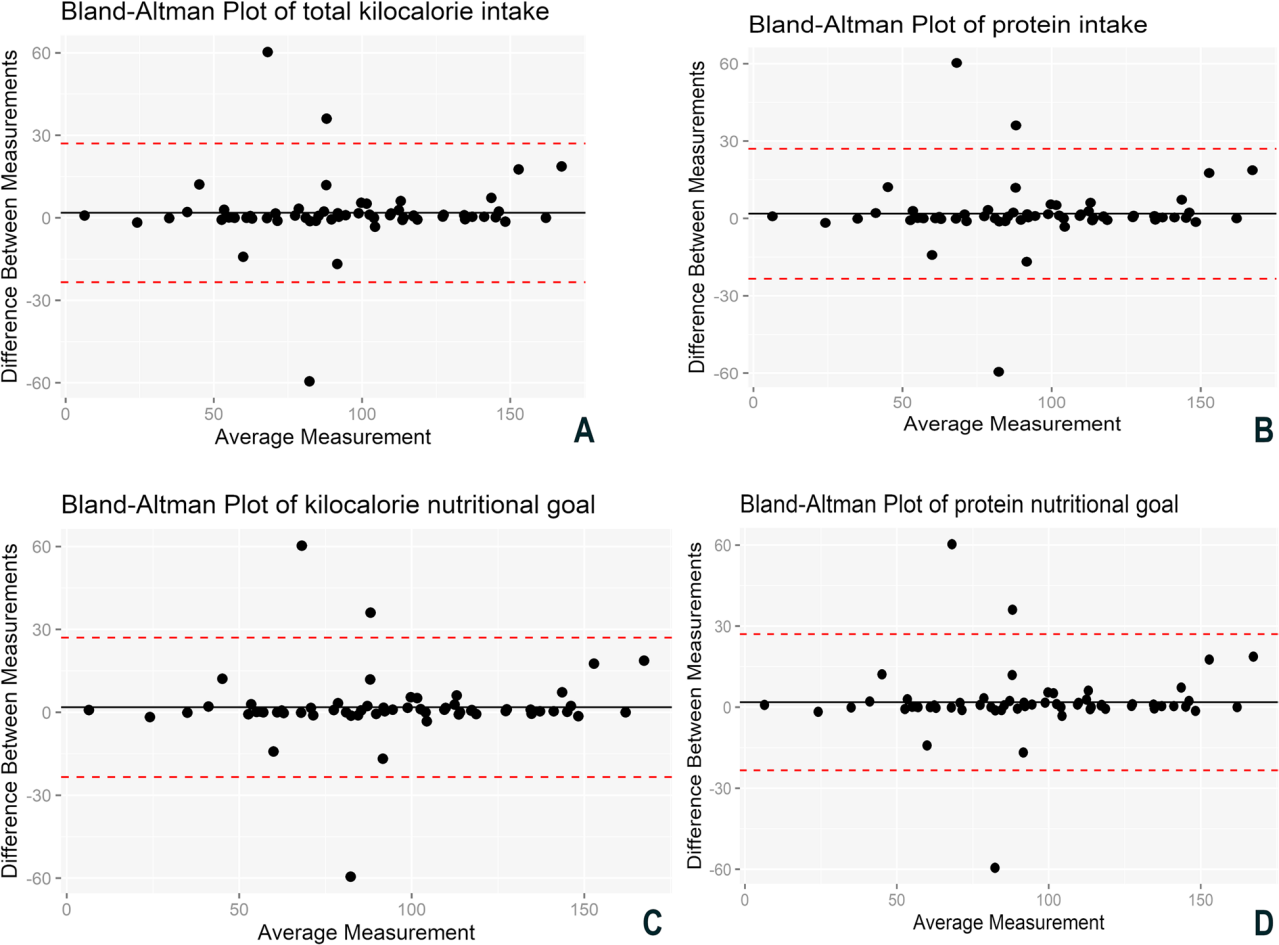
Bland–Altman plot of total kilocalorie intake (**A**), total protein intake (**B**), kilocalorie nutritional goal (**C**), and protein nutritional goal (**D**) measured by the paper instrument and the virtual application

The ARIETI showed good reliability for dietary anamnesis, as can be confirmed in the linear dispersion presented in Fig. 4A–D (Bland–Altman plots on the distribution and mean nutritional goal and calorie and protein intake goals, according to the paper instrument and ARIETI).

**Fig. 5:**
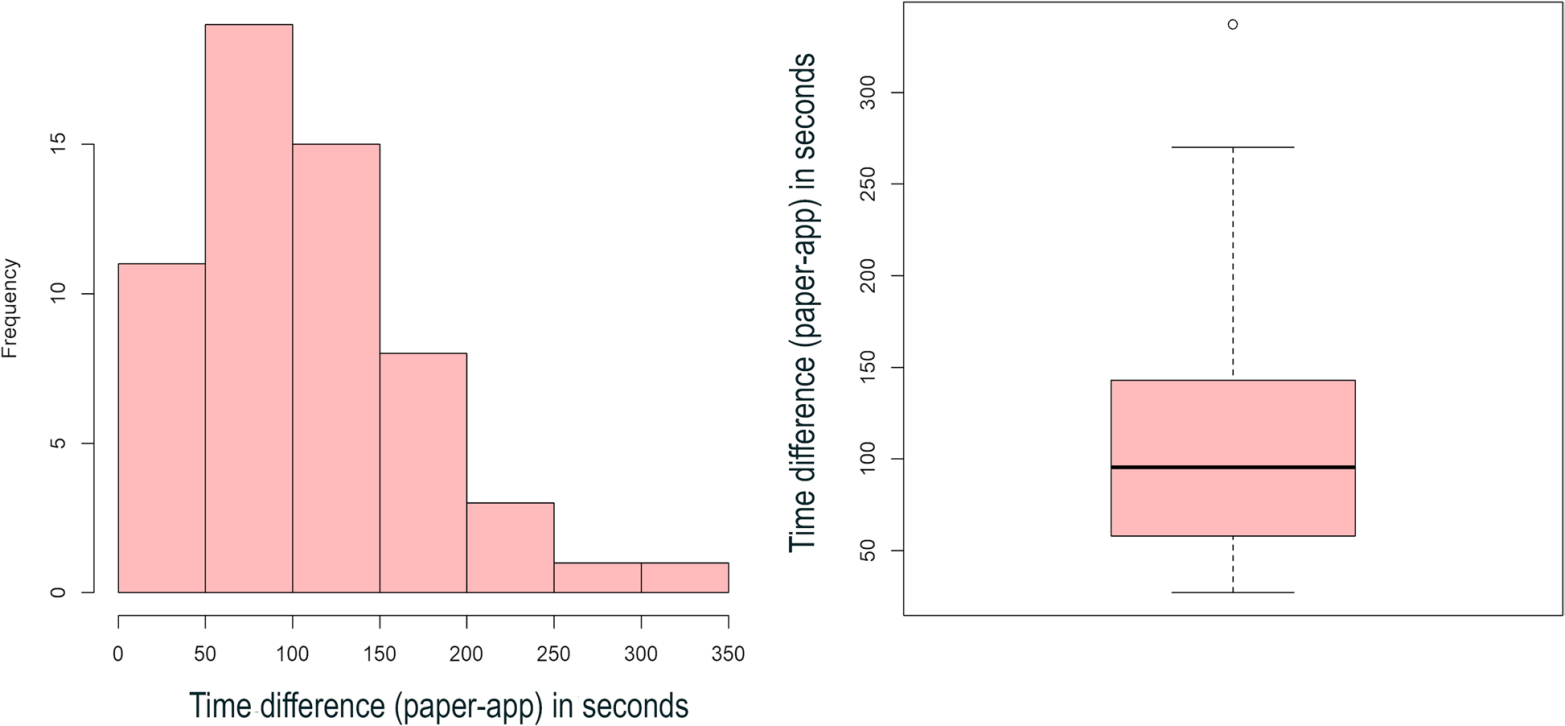
Analysis of the difference between decision-making times

The ARIETI accelerated the time required between administration and decision making by the nutrition professional according to the patient’s nutritional diagnosis by approximately 150 s (Fig. 5). The time required to perform all calculations and release the nutritional status diagnosis for each patient was 250 s longer than the app. This is because in the paper-based instrument, calculations are done manually (Fig. 5).

## Discussion

In this study, we aimed to demonstrate the development phases and reliability of the smartphone application ARIETI, a digital solution for in-hospital clinical nutritional practice. The decision-making process of the nutrition professional is crucial in maintaining the patient's nutritional status; therefore, the time to make this diagnosis should be as quick as possible to avoid negatively affecting their recovery.

A patient may experience adverse reactions to pharmacological treatment, making it difficult to eat and directly influencing the worsening of comorbidities or a deterioration in their health condition. Additionally, checking the dietary acceptance is equally important because in some cases, the patient may not eat properly due to personal dislikes for the food provided in the hospital environment, a situation that can be easily adjusted by the nutrition professional responsible to align the patient's preferences with the proposed nutritional therapy regimen. Therefore, the diagnosis of nutritional status and the assessment of hospital diet acceptance are important steps to be carried out during bedside care, a fact that the use of the mobile application developed in this study facilitates. Furthermore, it is worth noting the lack of applications with this purpose in the market.

The reliability analysis of the developed digital solution showed the following main findings: a) the ARIETI accelerated the time to identify nutritional risk associated with food intake compared to the paper-based instrument; b) the ARIETI was reliable and could replace the paper-based dietary anamnesis instrument that is currently validated for hospital settings.

The interactive and humanized model (Fig. 1) of the proposed instrument (ARIETI) optimizes dietary intake diagnostic activities, saves professional time, and avoids rework. The application was designed to be used intuitively, without requiring training or prior knowledge for its utilization. This makes it easy for other healthcare professionals to use, assisting in their daily routines and promoting multidisciplinary care. Additionally, this form of management contributes to the appropriate and agile adjustment of hospital diets and better monitoring of clinical nutritional progression.

Food intake assessment is crucial to the dietary management of inpatients [13]. Monitoring and recording the entire dietary content ingested by the patient are important components of their nutritional care, especially for those considered to be at higher nutritional risk [14, 15].

The gold standard for assessing the acceptance of hospital diet in clinical studies is the weighing of food remnants that are not ingested by patients [16–20]; however, this is often unfeasible in the routine assessment of inpatients, as it requires a longer time and a greater number of professionals. Therefore, the instrument validated by Silva [8] was compared with weighing as the basis for developing the ARIETI.

The time to administer the methods, which consists of completing the paper and virtual forms in the application, was equal because the completion time depended directly on the patient’s response time at the bedside. However, the time to make the necessary calculations for the nutritional diagnosis of the patient showed a slight difference (Fig. 5).

The difference between the time of calculation and the decision on the patient’s nutritional diagnosis is crucial considering that the virtual application provides the results of this analysis in real time at the bedside, fully streamlining this process, thereby having the potential to provide nutritional care actions with greater agility and in a shorter time interval [21].

In some cases, intercurrences, such as forgetting details of the anamnesis, may occur at the time of the clinical visit that influence the process of recording patient data before nutritional calculations. Given that the ARIETI returns data that have already been calculated in real time, its long-term use can save a considerable amount of time on calculations, in addition to ensuring that human errors do not occur in the diagnostic operation of nutritional risk related to in-hospital food intake capacity. Sometimes, the nutrition professional receives the nutritional consumption data from the patient 24 h after data collection, risking unfavorable clinical outcomes for the patient. The app streamlines the work of the clinical nutritionist by providing real-time analysis and calculations for nutritional diagnosis at the bedside, thereby bringing benefits to both the patient and the professional.

The ARIETI was developed to modernize the method and make it significantly more practical and rapid to accelerate data collection, with the aim of translating into continuous quality of care improvements to service users [22]. Storing data digitally saves space and reduces costs compared to storage using paper and other materials, in addition to enabling access from anywhere in the world [23]. Therefore, using an electronic system in the cloud, physical devices, or backups for nutritional data collection improves data security, reliability, and accessibility. Despite being validated in a study with hospitalized patients with infectious diseases, the application can also be tested in studies involving outpatient patients or even other populations such as pediatrics, geriatrics or oncology.

The curve tended to the right (Fig. 4), changing the normality of the distribution because the study included fewer patients with severe symptoms than with normal conditions (stabilized pathological condition). The severity of the condition affects the nutritional diagnosis given that patients with more severe disease tend to develop a nutritional deficit [24]. Given this characteristic, we used the Wilcoxon test to analyze the relationship between the two methods.

The boxplot shows the data distribution and outliers, thereby providing a complementary means to develop a better perspective on the characteristics of the dietary intake variables and nutritional goals. Additionally, the boxplot served as a comparative graphical layout of the two instruments. The discrete presence of outliers and the slight differences in the boxplot medians (Fig. 4) may theorize as a cause of possible human calculation and measurement errors with the paper instrument [25].

As an alternative to Student’s t test, in which the samples follow a normal distribution, the Wilcoxon test proved the similarity of the medians of the calorie and protein food intake variables, in addition to the nutritional goals obtained. Thus, it is possible to assess the efficacy of the virtual method tested, ensuring the safe assessment of the nutritional diagnosis of inpatients through food intake analysis and established nutritional goals [26].

The power of statistical significance by Pearson’s correlation < 0.001 (Table 2) for food intake and nutritional goals proves the linear trend of the positively correlated data given that the magnitudes remained similar according to the variations [26].

The Bland–Altman method demonstrated the dispersion between the individual means of food intake and nutritional goal variables, in addition to the individual differences between them, describing the agreement between the two methods (paper and ARIETI) considering quantitative variables. The assessment of the limits of agreement should include the clinical perspective, i.e., to determine whether the differences given by the limits can be considered acceptable [27]. The Bland–Altman plot was qualitatively employed to assess whether there is any difference in the trend of data distribution between the two instruments. These differences should be approximately independent to be valid.

The Bland–Altman method should be used in full when it is necessary to assess the agreement between two methods, including limits of agreement and their confidence intervals. If a change becomes essential, the data must be corrected to obtain a reliable and cohesive conclusion [28]. There was good dispersion of the data given that the results followed the trend lines of the means, and most of the distribution was within the limits of the 95% confidence intervals (Fig. 5A–D). The instruments did not exhibit significant differences in distribution trend, proving to be entirely independent. This once again confirms the consistency and reliability of the application when compared to the paper-based instrument.

### Key findings

The ARIETI demonstrated high reliability and efficacy as a virtual instrument to diagnose nutritional risk by dietary analysis and can therefore be reliably implemented in clinical practice. The application optimized the time between the diagnosis of nutritional risk related to dietary intake and the nutritionist’s decision making, improving the quality of information compared to the paper-based instrument.

### Limitations

The ARIETI requires internet access given the required communication with the relational database (SQL) and has been developed for the Android operating system (OS) versions 4.1 or higher; devices with other OS versions cannot run the developed software, as well as devices with very outdated Android systems.

Another important point is that the application was validated in a specific group of patients in a reference hospital for the treatment of infectious diseases (HIV, tuberculosis, trypanosomiasis, among others). Therefore, further studies are needed to expand its use to other patient groups.

## Conclusion

The ARIETI application has demonstrated equivalent reliability compared to the original instrument. Moreover, it optimized the time between the diagnosis of nutritional risk related to dietary intake and the nutritionist’s decision making, showing an improved ability to maintain information quality compared to the paper-based instrument. ARIETI reduced the difficulties and limitations of a paper instrument, such as loss and key element transcription or analysis errors in food intake control.

The use of technology focused on improving management, integrating information, and promoting efficiency gains allows health professionals to focus on their main objective, prescribing proper hospital diets and improving the efficiency of the therapeutic and nutritional processes in the hospital environment.

## Data Availability

All data generated or analyzed during this study are included in this published article.

## Abbreviations

App: Application
ARIETI: Mobile application to represent the food intake of inpatients
Diff: Difference
STD: Sexually transmitted diseases
H0: Null hypothesis
HIV: Human immunodeficiency virus
HTLV: Human cell lymphotropic virus
HTTPS: Hyper Text Transfer Protocol
INI: Infectious Diseases Institute
Kcal: Kilocalorie
Kcal goal: Nutritional total kilocalorie intake goal for the patient
Kcal total: Total kilocalories ingested by the patient
OS: Operating system
PTN: Protein
PTN Goal: Nutritional total protein intake goal for the patient
PTN total: Total protein ingested by the patient
Stat: Test statistics
TLS: Transport Layer Security
Var: Variable
